# *catena*-Poly[[[(3,5-dimethyl-1*H*-pyrazole)­copper(II)]-μ-{*N*-[1-(2-oxidophen­yl)ethyl­idene]-l-valinato}] methanol monosolvate]

**DOI:** 10.1107/S1600536811000304

**Published:** 2011-01-15

**Authors:** Gan-Qing Zhao, Xu-Dong Li, Yong-Jun Han, Ling-Wei Xue, Qin-Long Peng

**Affiliations:** a School of Chemistry and Chemical Engineering, Pingdingshan University, Pingdingshan 467000, People’s Republic of China

## Abstract

The asymmetric unit of the title compound, {[Cu(C_13_H_15_NO_3_)(C_5_H_8_N_2_)]·CH_3_OH}_*n*_, contains two complex mol­ecules and two solvent mol­ecules. Each Cu^II^ ion is in a distorted square-pyramidal coordination with one N and two O atoms from the Schiff base ligand and one N atom from the heterocycle in the basal positions and one carboxyl­ate O atom from a neighbouring ligand in the apical position. The apical Cu—O bonds are much longer than the basal Cu—O and Cu—N bonds. The carboxyl­ate groups of the Schiff base ligands bridge the Cu^II^ ions, forming helical chains along [100]. The crystal packing is stabilized by inter­molecular O—H⋯O and N—H⋯O hydrogen bonds.

## Related literature

For background to metal complexes with Schiff bases derived from amino acids, see: Basu Baul *et al.* (2007 ▶); Casella & Guillotti (1983 ▶); Ganguly *et al.* (2008 ▶); Parekh *et al.* (2006 ▶); Vigato & Tamburini (2004 ▶); Zhao *et al.* (2008 ▶, 2009 ▶). For synthetic details, see: Plesch *et al.* (1997 ▶).
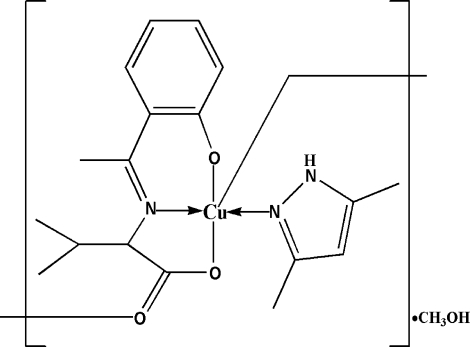


## Experimental

### 

#### Crystal data

[Cu(C_13_H_15_NO_3_)(C_5_H_8_N_2_)]·CH_4_O
*M* *_r_* = 424.98
Orthorhombic, 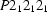

*a* = 14.12 (2) Å
*b* = 15.44 (2) Å
*c* = 21.25 (3) Å
*V* = 4634 (11) Å^3^
*Z* = 8
Mo *K*α radiation
μ = 0.97 mm^−1^
*T* = 296 K
0.25 × 0.21 × 0.17 mm

#### Data collection

Bruker APEXII CCD diffractometer
Absorption correction: multi-scan (*SADABS*; Bruker, 2008 ▶) *T*_min_ = 0.794, *T*_max_ = 0.853
23421 measured reflections
8496 independent reflections
4724 reflections with *I* > 2σ(*I*)
*R*_int_ = 0.071

#### Refinement

*R*[*F* ^2^ > 2σ(*F* ^2^)] = 0.054
*wR*(*F* ^2^) = 0.130
*S* = 1.01
8496 reflections
496 parameters
2 restraints
H-atom parameters constrained
Δρ_max_ = 0.49 e Å^−3^
Δρ_min_ = −0.31 e Å^−3^
Absolute structure: Flack (1983 ▶), 4318 Friedel pairs
Flack parameter: −0.012 (18)

### 

Data collection: *APEX2* (Bruker, 2008 ▶); cell refinement: *SAINT* (Bruker, 2008 ▶); data reduction: *SAINT*; program(s) used to solve structure: *SHELXS97* (Sheldrick, 2008 ▶); program(s) used to refine structure: *SHELXL97* (Sheldrick, 2008 ▶); molecular graphics: *SHELXTL* (Sheldrick, 2008 ▶); software used to prepare material for publication: *publCIF* (Westrip, 2010 ▶).

## Supplementary Material

Crystal structure: contains datablocks global, I. DOI: 10.1107/S1600536811000304/wm2441sup1.cif

Structure factors: contains datablocks I. DOI: 10.1107/S1600536811000304/wm2441Isup2.hkl

Additional supplementary materials:  crystallographic information; 3D view; checkCIF report

## Figures and Tables

**Table 1:** Selected bond lengths (Å)

Cu1—O1	1.899 (5)
Cu1—N1	1.978 (5)
Cu1—O2	1.989 (4)
Cu1—N5	2.027 (6)
Cu1—O6^i^	2.437 (6)
Cu2—O4	1.917 (5)
Cu2—N2	1.979 (5)
Cu2—O5	1.984 (4)
Cu2—N3	2.014 (5)
Cu2—O3	2.369 (5)

Symmetry code: (i) 
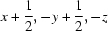
.

**Table 2:** Hydrogen-bond geometry (Å, °)

*D*—H⋯*A*	*D*—H	H⋯*A*	*D*⋯*A*	*D*—H⋯*A*
N6—H6*E*⋯O5^i^	0.86	2.01	2.843 (8)	164
N4—H4*E*⋯O2	0.86	2.06	2.873 (8)	157
O7—H7⋯O4	0.85	2.22	3.066 (14)	179
O8—H8*D*⋯O1^ii^	0.82	2.24	3.009 (12)	157

Symmetry codes: (i) 
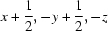
; (ii) 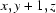.

**Fig. 1.**
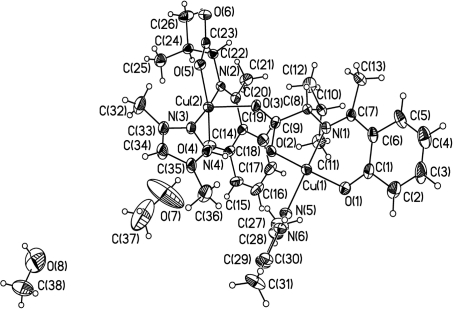
The molecular structure of the title compound, showing 50% probability displacement ellipsoids and the atom-numbering scheme.

**Fig. 2.**
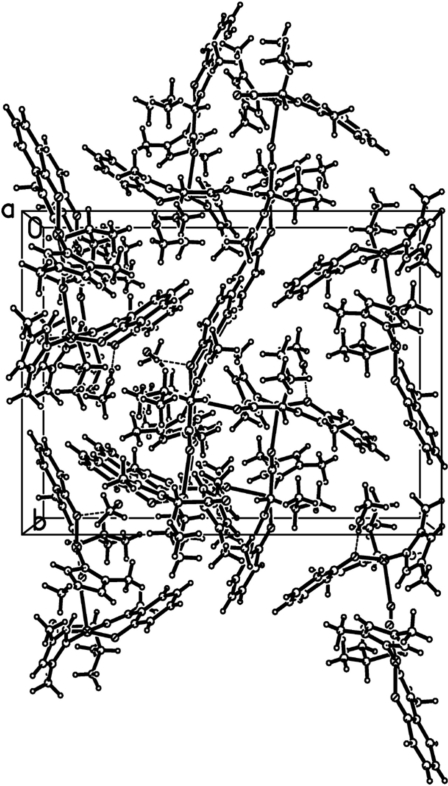
A view of the crystal packing viewed along the a axis.

## supplementary crystallographic information

### Comment

In the past decades, significant progress has been achieved in understanding the chemistry of transition metal complexes with Schiff base ligands composed of salicylaldehyde, 2-formylpyridine or their analogues, and α-amino acids (Vigato & Tamburini, 2004; Ganguly *et al.*, 2008; Casella & Guillotti, 1983). A few stuctural studies have been performed on Schiff base complexes derived from 2-hydroxyacetophenone and animo acids (Basu Baul *et al.*, 2007; Parekh *et al.*, 2006; Zhao *et al.*, 2008, 2009). We report here the crystal structure of the title Cu^II^ complex, [Cu(C_13_H_15_NO_3_)(C_5_H_8_N_2_)].CH_3_OH.

The asymmetric unit of the polymeric title compound consists of two Cu^II^ complex molecules and two solvate methanol molecules (Fig. 1). Each of the two Cu^II^ ions has a square-pyramidal coordination where the four basal positions are occupied by an O,N,O, donor set from the tridentate Schiff base ligand and the fourth position occupied by one N atom from the 3,5-dimethylpyrazole ligand. The apical position is occupied by a carboxylate O atom from the adjacent tridentate Schiff base ligand. The apical Cu···O bonds are much longer than the basal Cu···O and Cu···N bonds (Table 1). The closest distance between neighbouring Cu^II^ ions are 5.803 (6) Å and 5.890 (6) Å, respectively.

The crystal structure is stabilized by intermolecular N—H···O hydrogen bonds between the pyrazole N—H groups as donors and the carboxylate O atoms as acceptors. Additional O—H···O hydrogen bonding involving the methanol solvent molecules is also observed (Table 2 and Fig. 2).

### Experimental

The title compound was synthesized as described in the literature (Plesch *et al.*, 1997). To L-valine (1.00 mmol) and potassium hydroxide (1.00 mmol) in 10 ml of methanol was added 2-hydroxyacetophenone (1.00 mmol in 10 ml of methanol) dropwise. The yellow solution was stirred for 2.0 h at 333 K. The resultant mixture was added dropwise to copper(II) acetate monohydrate (1.00 mmol) and 3,5-dimethylpyrazole (1.00 mmol) in an aqueous methanolic solution (20 ml, 1:1 *v*/*v*), and heated with stirring for 2.0 h at 333 K. The dark blue solution was filtered and left for several days. Blue crystals had formed that were filtered off, washed with water, and dried under vacuum.

### Refinement

All H atoms were positioned geometrically and refined as riding atoms, with C—H = 0.93 or 0.98 Å (CH) and *U*_iso_(H) = 1.2*U*_eq_(C), C—H = 0.96 Å (CH_3_) and *U*_iso_(H) = 1.5*U*_eq_(C), with N—H = 0.86 Å and *U*_iso_(H) = 1.2*U*_eq_(N), and with O—H = 0.82 Å and 0.85 Å and *U*_iso_(H) = 1.5*U*_eq_(O).

### Crystal data

**Table:**

[Cu(C_13_H_15_NO_3_)(C_5_H_8_N_2_)]·CH_4_O	*F*(000) = 1784
*M**_r_* = 424.98	*D*_x_ = 1.218 Mg m^−^^3^
Orthorhombic, *P*2_1_2_1_2_1_	Mo *K*α radiation, λ = 0.71073 Å
Hall symbol: P 2ac 2ab	Cell parameters from 3320 reflections
*a* = 14.12 (2) Å	θ = 2.3–17.8°
*b* = 15.44 (2) Å	µ = 0.97 mm^−^^1^
*c* = 21.25 (3) Å	*T* = 296 K
*V* = 4634 (11) Å^3^	Block, blue
*Z* = 8	0.25 × 0.21 × 0.17 mm

### Data collection

**Table:**

Bruker APEXII CCD diffractometer	8496 independent reflections
Radiation source: fine-focus sealed tube	4724 reflections with *I* > 2σ(*I*)
graphite	*R*_int_ = 0.071
φ and ω scans	θ_max_ = 25.5°, θ_min_ = 2.3°
Absorption correction: multi-scan (*SADABS*; Bruker, 2008)	*h* = −17→17
*T*_min_ = 0.794, *T*_max_ = 0.853	*k* = −18→16
23421 measured reflections	*l* = −25→25

### Refinement

**Table:**

Refinement on *F*^2^	Secondary atom site location: difference Fourier map
Least-squares matrix: full	Hydrogen site location: inferred from neighbouring sites
*R*[*F*^2^ > 2σ(*F*^2^)] = 0.054	H-atom parameters constrained
*wR*(*F*^2^) = 0.130	*w* = 1/[σ^2^(*F*_o_^2^) + (0.0525*P*)^2^] where *P* = (*F*_o_^2^ + 2*F*_c_^2^)/3
*S* = 1.01	(Δ/σ)_max_ = 0.001
8496 reflections	Δρ_max_ = 0.49 e Å^−^^3^
496 parameters	Δρ_min_ = −0.31 e Å^−^^3^
2 restraints	Absolute structure: Flack (1983), 4318 Friedel pairs
Primary atom site location: structure-invariant direct methods	Flack parameter: −0.012 (18)

### Special details

**Table:**

Geometry. All e.s.d.'s (except the e.s.d. in the dihedral angle between two l.s. planes) are estimated using the full covariance matrix. The cell e.s.d.'s are taken into account individually in the estimation of e.s.d.'s in distances, angles and torsion angles; correlations between e.s.d.'s in cell parameters are only used when they are defined by crystal symmetry. An approximate (isotropic) treatment of cell e.s.d.'s is used for estimating e.s.d.'s involving l.s. planes.
Refinement. Refinement of *F*^2^ against ALL reflections. The weighted *R*-factor *wR* and goodness of fit *S* are based on *F*^2^, conventional *R*-factors *R* are based on *F*, with *F* set to zero for negative *F*^2^. The threshold expression of *F*^2^ > σ(*F*^2^) is used only for calculating *R*-factors(gt) *etc*. and is not relevant to the choice of reflections for refinement. *R*-factors based on *F*^2^ are statistically about twice as large as those based on *F*, and *R*- factors based on ALL data will be even larger.

### Fractional atomic coordinates and isotropic or equivalent isotropic displacement parameters (Å^2^)

**Table:**

	*x*	*y*	*z*	*U*_iso_*/*U*_eq_	
Cu1	0.25158 (5)	0.08464 (4)	0.09864 (3)	0.0529 (2)	
Cu2	0.00319 (5)	0.39015 (4)	0.11651 (3)	0.0523 (2)	
O1	0.3151 (3)	−0.0237 (3)	0.1022 (2)	0.0763 (14)	
O2	0.1746 (2)	0.1914 (2)	0.11032 (19)	0.0536 (10)	
O3	0.0238 (3)	0.2396 (3)	0.1001 (2)	0.0641 (12)	
O4	0.0751 (3)	0.4047 (3)	0.1923 (2)	0.0670 (13)	
O5	−0.0818 (2)	0.3982 (3)	0.04239 (17)	0.0548 (11)	
O6	−0.2349 (3)	0.3840 (3)	0.01337 (19)	0.0655 (12)	
O8	0.4396 (9)	0.9321 (8)	0.2125 (4)	0.249 (5)	
H8D	0.4058	0.9581	0.1874	0.374*	
O7	0.2448 (12)	0.5282 (14)	0.1852 (6)	0.441 (13)	
H7	0.1976	0.4942	0.1873	0.662*	
N1	0.1280 (3)	0.0283 (3)	0.0830 (2)	0.0484 (13)	
N2	−0.1155 (3)	0.3732 (3)	0.1645 (2)	0.0488 (13)	
N3	0.1192 (3)	0.4188 (3)	0.0654 (2)	0.0579 (14)	
N4	0.1979 (3)	0.3673 (3)	0.0691 (2)	0.0573 (15)	
H4E	0.2001	0.3200	0.0904	0.069*	
N5	0.3720 (3)	0.1457 (3)	0.1255 (3)	0.0578 (14)	
N6	0.4471 (3)	0.1506 (3)	0.0852 (2)	0.0589 (15)	
H6E	0.4478	0.1298	0.0476	0.071*	
C1	0.2882 (5)	−0.0935 (5)	0.0706 (3)	0.0666 (19)	
C2	0.3572 (5)	−0.1620 (6)	0.0619 (4)	0.096 (3)	
H2	0.4172	−0.1566	0.0795	0.115*	
C3	0.3344 (7)	−0.2360 (6)	0.0273 (6)	0.120 (4)	
H3	0.3806	−0.2783	0.0219	0.144*	
C4	0.2467 (8)	−0.2491 (6)	0.0009 (5)	0.114 (3)	
H4	0.2342	−0.2984	−0.0228	0.136*	
C5	0.1762 (6)	−0.1862 (5)	0.0105 (4)	0.086 (2)	
H5	0.1161	−0.1959	−0.0059	0.103*	
C6	0.1934 (5)	−0.1082 (4)	0.0445 (3)	0.0621 (18)	
C7	0.1147 (4)	−0.0466 (4)	0.0553 (3)	0.0505 (16)	
C8	0.0473 (4)	0.0867 (4)	0.0983 (3)	0.0473 (14)	
H8	0.0013	0.0834	0.0639	0.057*	
C9	0.0830 (4)	0.1800 (4)	0.1027 (3)	0.0526 (16)	
C10	−0.0032 (5)	0.0571 (4)	0.1607 (3)	0.0592 (16)	
H10	−0.0113	−0.0058	0.1580	0.071*	
C11	0.0586 (5)	0.0748 (5)	0.2189 (3)	0.084 (2)	
H11A	0.0705	0.1358	0.2223	0.127*	
H11B	0.1177	0.0445	0.2147	0.127*	
H11C	0.0263	0.0550	0.2559	0.127*	
C12	−0.1034 (4)	0.0967 (5)	0.1674 (4)	0.091 (2)	
H12A	−0.1301	0.0800	0.2072	0.137*	
H12B	−0.1430	0.0759	0.1340	0.137*	
H12C	−0.0992	0.1587	0.1653	0.137*	
C13	0.0147 (4)	−0.0743 (4)	0.0329 (3)	0.077 (2)	
H13A	−0.0319	−0.0367	0.0511	0.115*	
H13B	0.0027	−0.1329	0.0458	0.115*	
H13C	0.0114	−0.0706	−0.0122	0.115*	
C14	0.0568 (5)	0.3587 (5)	0.2436 (3)	0.065 (2)	
C15	0.1329 (5)	0.3434 (5)	0.2870 (4)	0.077 (2)	
H15	0.1914	0.3690	0.2792	0.092*	
C16	0.1225 (7)	0.2919 (6)	0.3399 (4)	0.093 (3)	
H16	0.1747	0.2812	0.3654	0.111*	
C17	0.0353 (7)	0.2557 (6)	0.3556 (4)	0.092 (3)	
H17	0.0280	0.2214	0.3912	0.111*	
C18	−0.0402 (5)	0.2732 (5)	0.3158 (4)	0.080 (2)	
H18	−0.0986	0.2492	0.3261	0.096*	
C19	−0.0355 (5)	0.3254 (4)	0.2598 (3)	0.0561 (18)	
C20	−0.1224 (5)	0.3417 (4)	0.2217 (3)	0.0562 (17)	
C21	−0.2190 (4)	0.3199 (5)	0.2517 (3)	0.084 (2)	
H21A	−0.2687	0.3462	0.2275	0.126*	
H21B	−0.2208	0.3417	0.2940	0.126*	
H21C	−0.2276	0.2582	0.2522	0.126*	
C22	−0.2019 (4)	0.3908 (4)	0.1250 (3)	0.0514 (16)	
H22	−0.2482	0.3446	0.1322	0.062*	
C23	−0.1728 (4)	0.3904 (4)	0.0544 (3)	0.0483 (16)	
C24	−0.2478 (5)	0.4798 (4)	0.1436 (3)	0.0593 (17)	
H24	−0.2498	0.4818	0.1896	0.071*	
C25	−0.1882 (5)	0.5574 (5)	0.1217 (4)	0.085 (2)	
H25A	−0.1244	0.5508	0.1364	0.127*	
H25B	−0.2146	0.6098	0.1385	0.127*	
H25C	−0.1885	0.5600	0.0766	0.127*	
C26	−0.3513 (5)	0.4860 (5)	0.1202 (4)	0.098 (3)	
H26A	−0.3761	0.5423	0.1298	0.147*	
H26B	−0.3889	0.4425	0.1407	0.147*	
H26C	−0.3530	0.4769	0.0755	0.147*	
C27	0.3314 (5)	0.1887 (6)	0.2353 (3)	0.105 (3)	
H27A	0.2788	0.2261	0.2263	0.157*	
H27B	0.3655	0.2107	0.2710	0.157*	
H27C	0.3085	0.1316	0.2445	0.157*	
C28	0.3973 (5)	0.1852 (5)	0.1781 (3)	0.070 (2)	
C29	0.4918 (5)	0.2168 (5)	0.1726 (3)	0.083 (2)	
H29	0.5264	0.2470	0.2027	0.100*	
C30	0.5211 (4)	0.1931 (4)	0.1128 (4)	0.0708 (19)	
C31	0.6134 (5)	0.2107 (6)	0.0764 (4)	0.116 (3)	
H31A	0.6236	0.2720	0.0735	0.174*	
H31B	0.6087	0.1866	0.0349	0.174*	
H31C	0.6656	0.1844	0.0982	0.174*	
C32	0.0749 (6)	0.5596 (5)	0.0159 (5)	0.119 (3)	
H32A	0.0122	0.5356	0.0136	0.178*	
H32B	0.0905	0.5862	−0.0236	0.178*	
H32C	0.0773	0.6023	0.0487	0.178*	
C33	0.1462 (5)	0.4871 (5)	0.0302 (4)	0.077 (2)	
C34	0.2404 (5)	0.4760 (5)	0.0111 (4)	0.094 (3)	
H34	0.2756	0.5138	−0.0137	0.113*	
C35	0.2713 (5)	0.3982 (5)	0.0360 (4)	0.078 (2)	
C36	0.3655 (5)	0.3518 (5)	0.0341 (5)	0.116 (3)	
H36A	0.3769	0.3244	0.0740	0.174*	
H36B	0.4150	0.3927	0.0256	0.174*	
H36C	0.3644	0.3086	0.0016	0.174*	
C37	0.3268 (13)	0.5866 (11)	0.1693 (9)	0.362 (15)	
H37A	0.3451	0.5776	0.1263	0.544*	
H37B	0.3793	0.5735	0.1964	0.544*	
H37C	0.3083	0.6459	0.1751	0.544*	
C38	0.5428 (9)	0.9492 (12)	0.1950 (9)	0.341 (15)	
H38A	0.5767	0.9684	0.2316	0.512*	
H38B	0.5455	0.9932	0.1631	0.512*	
H38C	0.5709	0.8969	0.1794	0.512*	

### Atomic displacement parameters (Å^2^)

**Table:**

	*U*^11^	*U*^22^	*U*^33^	*U*^12^	*U*^13^	*U*^23^
Cu1	0.0443 (4)	0.0502 (4)	0.0643 (5)	−0.0002 (4)	−0.0002 (4)	−0.0025 (4)
Cu2	0.0433 (4)	0.0560 (4)	0.0576 (4)	−0.0004 (4)	0.0002 (4)	0.0002 (4)
O1	0.065 (3)	0.055 (3)	0.109 (4)	0.011 (2)	−0.011 (3)	−0.009 (3)
O2	0.046 (2)	0.046 (2)	0.068 (3)	−0.0055 (18)	0.004 (2)	−0.002 (2)
O3	0.059 (3)	0.043 (2)	0.090 (3)	0.010 (2)	0.000 (2)	0.006 (2)
O4	0.051 (2)	0.092 (4)	0.058 (3)	−0.007 (3)	−0.006 (2)	0.001 (3)
O5	0.044 (2)	0.068 (3)	0.052 (2)	0.006 (2)	0.0053 (18)	0.004 (2)
O6	0.050 (2)	0.087 (3)	0.059 (3)	−0.006 (3)	−0.009 (2)	−0.003 (2)
O8	0.336 (14)	0.283 (13)	0.128 (7)	−0.032 (13)	−0.011 (8)	0.050 (8)
O7	0.420 (19)	0.72 (3)	0.182 (11)	−0.36 (2)	0.066 (11)	−0.203 (16)
N1	0.053 (3)	0.039 (3)	0.053 (3)	−0.001 (2)	0.007 (3)	0.001 (3)
N2	0.047 (3)	0.046 (3)	0.053 (3)	−0.002 (2)	−0.004 (2)	−0.002 (3)
N3	0.052 (3)	0.046 (3)	0.076 (4)	0.003 (3)	0.006 (3)	0.009 (3)
N4	0.051 (3)	0.055 (4)	0.066 (4)	0.002 (3)	0.005 (3)	0.020 (3)
N5	0.050 (3)	0.067 (4)	0.057 (4)	−0.004 (3)	0.002 (3)	−0.007 (3)
N6	0.046 (3)	0.074 (4)	0.057 (4)	−0.009 (3)	−0.006 (3)	0.001 (3)
C1	0.068 (4)	0.047 (5)	0.084 (5)	0.009 (4)	0.021 (4)	0.014 (4)
C2	0.069 (5)	0.081 (6)	0.138 (8)	0.014 (5)	0.033 (5)	0.024 (6)
C3	0.112 (8)	0.052 (6)	0.195 (12)	0.018 (6)	0.058 (8)	−0.017 (7)
C4	0.123 (7)	0.072 (5)	0.146 (8)	−0.005 (6)	0.062 (8)	−0.027 (5)
C5	0.098 (6)	0.061 (5)	0.099 (6)	−0.025 (5)	0.036 (5)	−0.032 (5)
C6	0.073 (4)	0.046 (4)	0.068 (4)	−0.006 (4)	0.029 (4)	0.004 (4)
C7	0.053 (4)	0.043 (4)	0.055 (4)	−0.004 (3)	0.009 (3)	0.008 (3)
C8	0.045 (3)	0.044 (4)	0.053 (4)	−0.002 (3)	−0.002 (3)	0.002 (4)
C9	0.063 (4)	0.047 (4)	0.048 (4)	0.004 (3)	0.004 (3)	0.006 (4)
C10	0.059 (4)	0.054 (4)	0.065 (4)	−0.013 (4)	0.006 (4)	0.000 (3)
C11	0.099 (6)	0.094 (6)	0.061 (5)	−0.005 (5)	0.010 (4)	0.005 (5)
C12	0.067 (5)	0.096 (6)	0.110 (6)	0.010 (5)	0.037 (4)	0.002 (6)
C13	0.072 (5)	0.069 (4)	0.089 (5)	−0.021 (4)	−0.006 (4)	−0.010 (4)
C14	0.072 (5)	0.071 (5)	0.051 (5)	0.021 (4)	−0.012 (4)	−0.007 (4)
C15	0.053 (4)	0.092 (6)	0.085 (6)	0.015 (4)	−0.017 (4)	−0.017 (5)
C16	0.106 (7)	0.092 (7)	0.080 (6)	0.046 (6)	−0.028 (6)	−0.006 (6)
C17	0.113 (7)	0.085 (7)	0.079 (6)	0.037 (6)	−0.021 (6)	0.003 (5)
C18	0.078 (5)	0.077 (6)	0.085 (6)	0.009 (4)	0.007 (5)	−0.008 (5)
C19	0.062 (4)	0.058 (5)	0.048 (4)	0.011 (3)	−0.004 (3)	0.000 (4)
C20	0.070 (5)	0.054 (4)	0.044 (4)	0.005 (4)	0.000 (3)	−0.004 (3)
C21	0.061 (5)	0.113 (6)	0.077 (5)	−0.003 (4)	0.011 (4)	0.026 (5)
C22	0.041 (3)	0.060 (4)	0.053 (4)	−0.004 (3)	−0.002 (3)	0.006 (4)
C23	0.050 (4)	0.041 (4)	0.054 (4)	0.001 (3)	0.007 (3)	−0.001 (4)
C24	0.060 (4)	0.056 (4)	0.062 (4)	0.014 (4)	−0.011 (4)	−0.009 (3)
C25	0.091 (5)	0.072 (5)	0.091 (6)	0.011 (4)	−0.006 (5)	−0.006 (5)
C26	0.057 (4)	0.123 (7)	0.113 (7)	0.038 (4)	−0.011 (5)	−0.010 (6)
C27	0.096 (6)	0.147 (8)	0.071 (5)	−0.009 (6)	0.014 (5)	−0.035 (6)
C28	0.060 (4)	0.097 (6)	0.052 (5)	−0.011 (4)	0.001 (4)	−0.013 (4)
C29	0.075 (5)	0.102 (6)	0.071 (5)	−0.013 (5)	−0.016 (5)	−0.031 (4)
C30	0.053 (4)	0.079 (5)	0.081 (5)	−0.012 (3)	−0.005 (4)	−0.014 (5)
C31	0.067 (5)	0.151 (9)	0.130 (8)	−0.042 (5)	0.015 (5)	−0.062 (7)
C32	0.087 (6)	0.092 (7)	0.177 (9)	0.024 (5)	0.025 (6)	0.060 (7)
C33	0.058 (4)	0.057 (5)	0.115 (7)	0.005 (4)	0.006 (4)	0.017 (5)
C34	0.063 (5)	0.073 (5)	0.147 (7)	−0.006 (5)	0.029 (6)	0.039 (5)
C35	0.065 (5)	0.061 (5)	0.109 (6)	−0.009 (4)	0.019 (4)	0.034 (5)
C36	0.066 (5)	0.113 (7)	0.170 (9)	0.016 (5)	0.037 (5)	0.059 (7)
C37	0.61 (5)	0.23 (2)	0.25 (2)	0.16 (3)	−0.13 (3)	−0.019 (18)
C38	0.29 (2)	0.246 (19)	0.49 (3)	−0.122 (17)	−0.29 (2)	0.06 (2)

### Geometric parameters (Å, °)

**Table:**

Cu1—O1	1.899 (5)	C13—H13B	0.9600
Cu1—N1	1.978 (5)	C13—H13C	0.9600
Cu1—O2	1.989 (4)	C14—C15	1.435 (9)
Cu1—N5	2.027 (6)	C14—C19	1.443 (9)
Cu1—O6^i^	2.437 (6)	C15—C16	1.386 (10)
Cu2—O4	1.917 (5)	C15—H15	0.9300
Cu2—N2	1.979 (5)	C16—C17	1.392 (11)
Cu2—O5	1.984 (4)	C16—H16	0.9300
Cu2—N3	2.014 (5)	C17—C18	1.388 (9)
Cu2—O3	2.369 (5)	C17—H17	0.9300
O1—C1	1.327 (8)	C18—C19	1.438 (10)
O2—C9	1.316 (7)	C18—H18	0.9300
O3—C9	1.244 (7)	C19—C20	1.492 (9)
O4—C14	1.328 (8)	C20—C21	1.543 (9)
O5—C23	1.316 (6)	C21—H21A	0.9600
O6—C23	1.241 (7)	C21—H21B	0.9600
O6—Cu1^ii^	2.437 (6)	C21—H21C	0.9600
O8—C38	1.526 (9)	C22—C23	1.555 (8)
O8—H8D	0.8200	C22—C24	1.568 (8)
O7—C37	1.506 (10)	C22—H22	0.9800
O7—H7	0.8495	C24—C25	1.536 (9)
N1—C7	1.311 (7)	C24—C26	1.546 (9)
N1—C8	1.488 (7)	C24—H24	0.9800
N2—C20	1.314 (7)	C25—H25A	0.9600
N2—C22	1.506 (7)	C25—H25B	0.9600
N3—C33	1.349 (8)	C25—H25C	0.9600
N3—N4	1.369 (7)	C26—H26A	0.9600
N4—C35	1.341 (7)	C26—H26B	0.9600
N4—H4E	0.8600	C26—H26C	0.9600
N5—C28	1.324 (8)	C27—C28	1.533 (9)
N5—N6	1.365 (7)	C27—H27A	0.9600
N6—C30	1.366 (7)	C27—H27B	0.9600
N6—H6E	0.8600	C27—H27C	0.9600
C1—C2	1.451 (10)	C28—C29	1.426 (9)
C1—C6	1.467 (9)	C29—C30	1.384 (9)
C2—C3	1.396 (12)	C29—H29	0.9300
C2—H2	0.9300	C30—C31	1.541 (9)
C3—C4	1.376 (13)	C31—H31A	0.9600
C3—H3	0.9300	C31—H31B	0.9600
C4—C5	1.405 (11)	C31—H31C	0.9600
C4—H4	0.9300	C32—C33	1.535 (10)
C5—C6	1.425 (9)	C32—H32A	0.9600
C5—H5	0.9300	C32—H32B	0.9600
C6—C7	1.480 (9)	C32—H32C	0.9600
C7—C13	1.551 (8)	C33—C34	1.402 (10)
C8—C9	1.529 (8)	C34—C35	1.382 (9)
C8—C10	1.574 (8)	C34—H34	0.9300
C8—H8	0.9800	C35—C36	1.512 (9)
C10—C11	1.537 (9)	C36—H36A	0.9600
C10—C12	1.548 (9)	C36—H36B	0.9600
C10—H10	0.9800	C36—H36C	0.9600
C11—H11A	0.9600	C37—H37A	0.9600
C11—H11B	0.9600	C37—H37B	0.9600
C11—H11C	0.9600	C37—H37C	0.9600
C12—H12A	0.9600	C38—H38A	0.9600
C12—H12B	0.9600	C38—H38B	0.9600
C12—H12C	0.9600	C38—H38C	0.9600
C13—H13A	0.9600		
			
O1—Cu1—N1	92.1 (2)	C16—C15—H15	118.8
O1—Cu1—O2	169.2 (2)	C14—C15—H15	118.8
N1—Cu1—O2	84.4 (2)	C15—C16—C17	121.2 (8)
O1—Cu1—N5	90.1 (2)	C15—C16—H16	119.4
N1—Cu1—N5	172.9 (2)	C17—C16—H16	119.4
O2—Cu1—N5	92.2 (2)	C18—C17—C16	117.1 (8)
O1—Cu1—O6^i^	100.21 (19)	C18—C17—H17	121.4
N1—Cu1—O6^i^	89.58 (17)	C16—C17—H17	121.4
O2—Cu1—O6^i^	90.00 (16)	C17—C18—C19	125.3 (7)
N5—Cu1—O6^i^	96.69 (19)	C17—C18—H18	117.4
O4—Cu2—N2	91.8 (2)	C19—C18—H18	117.4
O4—Cu2—O5	168.51 (19)	C18—C19—C14	116.0 (6)
N2—Cu2—O5	84.5 (2)	C18—C19—C20	120.4 (6)
O4—Cu2—N3	89.8 (2)	C14—C19—C20	123.6 (6)
N2—Cu2—N3	174.5 (2)	N2—C20—C19	120.3 (6)
O5—Cu2—N3	92.9 (2)	N2—C20—C21	121.9 (6)
O4—Cu2—O3	100.00 (19)	C19—C20—C21	117.8 (6)
N2—Cu2—O3	92.86 (17)	C20—C21—H21A	109.5
O5—Cu2—O3	91.09 (17)	C20—C21—H21B	109.5
N3—Cu2—O3	92.08 (19)	H21A—C21—H21B	109.5
C1—O1—Cu1	124.0 (4)	C20—C21—H21C	109.5
C9—O2—Cu1	114.3 (4)	H21A—C21—H21C	109.5
C9—O3—Cu2	143.2 (4)	H21B—C21—H21C	109.5
C14—O4—Cu2	121.6 (4)	N2—C22—C23	108.8 (4)
C23—O5—Cu2	115.5 (4)	N2—C22—C24	110.7 (5)
C23—O6—Cu1^ii^	136.6 (4)	C23—C22—C24	110.9 (5)
C38—O8—H8D	108.2	N2—C22—H22	108.8
C37—O7—H7	170.2	C23—C22—H22	108.8
C7—N1—C8	121.5 (5)	C24—C22—H22	108.8
C7—N1—Cu1	126.1 (4)	O6—C23—O5	124.1 (5)
C8—N1—Cu1	111.9 (4)	O6—C23—C22	119.5 (5)
C20—N2—C22	121.5 (5)	O5—C23—C22	116.5 (5)
C20—N2—Cu2	126.1 (4)	C25—C24—C26	111.8 (5)
C22—N2—Cu2	112.1 (4)	C25—C24—C22	112.4 (5)
C33—N3—N4	104.8 (5)	C26—C24—C22	111.3 (5)
C33—N3—Cu2	134.5 (4)	C25—C24—H24	107.0
N4—N3—Cu2	120.2 (4)	C26—C24—H24	107.0
C35—N4—N3	113.0 (5)	C22—C24—H24	107.0
C35—N4—H4E	123.5	C24—C25—H25A	109.5
N3—N4—H4E	123.5	C24—C25—H25B	109.5
C28—N5—N6	107.2 (5)	H25A—C25—H25B	109.5
C28—N5—Cu1	132.7 (4)	C24—C25—H25C	109.5
N6—N5—Cu1	120.1 (4)	H25A—C25—H25C	109.5
N5—N6—C30	110.5 (5)	H25B—C25—H25C	109.5
N5—N6—H6E	124.7	C24—C26—H26A	109.5
C30—N6—H6E	124.7	C24—C26—H26B	109.5
O1—C1—C2	117.7 (7)	H26A—C26—H26B	109.5
O1—C1—C6	125.4 (6)	C24—C26—H26C	109.5
C2—C1—C6	116.9 (8)	H26A—C26—H26C	109.5
C3—C2—C1	120.6 (8)	H26B—C26—H26C	109.5
C3—C2—H2	119.7	C28—C27—H27A	109.5
C1—C2—H2	119.7	C28—C27—H27B	109.5
C4—C3—C2	122.8 (9)	H27A—C27—H27B	109.5
C4—C3—H3	118.6	C28—C27—H27C	109.5
C2—C3—H3	118.6	H27A—C27—H27C	109.5
C3—C4—C5	118.5 (9)	H27B—C27—H27C	109.5
C3—C4—H4	120.8	N5—C28—C29	109.9 (6)
C5—C4—H4	120.8	N5—C28—C27	121.5 (6)
C4—C5—C6	122.5 (8)	C29—C28—C27	128.5 (6)
C4—C5—H5	118.7	C30—C29—C28	105.4 (6)
C6—C5—H5	118.7	C30—C29—H29	127.3
C5—C6—C1	118.5 (7)	C28—C29—H29	127.3
C5—C6—C7	119.6 (7)	N6—C30—C29	107.1 (6)
C1—C6—C7	121.8 (6)	N6—C30—C31	121.1 (7)
N1—C7—C6	121.9 (6)	C29—C30—C31	131.8 (7)
N1—C7—C13	120.8 (5)	C30—C31—H31A	109.5
C6—C7—C13	117.3 (6)	C30—C31—H31B	109.5
N1—C8—C9	109.4 (4)	H31A—C31—H31B	109.5
N1—C8—C10	110.8 (5)	C30—C31—H31C	109.5
C9—C8—C10	111.8 (5)	H31A—C31—H31C	109.5
N1—C8—H8	108.2	H31B—C31—H31C	109.5
C9—C8—H8	108.2	C33—C32—H32A	109.5
C10—C8—H8	108.2	C33—C32—H32B	109.5
O3—C9—O2	124.6 (6)	H32A—C32—H32B	109.5
O3—C9—C8	118.2 (5)	C33—C32—H32C	109.5
O2—C9—C8	117.2 (5)	H32A—C32—H32C	109.5
C11—C10—C12	112.0 (6)	H32B—C32—H32C	109.5
C11—C10—C8	111.6 (5)	N3—C33—C34	109.5 (6)
C12—C10—C8	112.2 (5)	N3—C33—C32	119.6 (6)
C11—C10—H10	106.9	C34—C33—C32	130.9 (7)
C12—C10—H10	106.9	C35—C34—C33	107.2 (6)
C8—C10—H10	106.9	C35—C34—H34	126.4
C10—C11—H11A	109.5	C33—C34—H34	126.4
C10—C11—H11B	109.5	N4—C35—C34	105.5 (6)
H11A—C11—H11B	109.5	N4—C35—C36	121.6 (6)
C10—C11—H11C	109.5	C34—C35—C36	132.8 (6)
H11A—C11—H11C	109.5	C35—C36—H36A	109.5
H11B—C11—H11C	109.5	C35—C36—H36B	109.5
C10—C12—H12A	109.5	H36A—C36—H36B	109.5
C10—C12—H12B	109.5	C35—C36—H36C	109.5
H12A—C12—H12B	109.5	H36A—C36—H36C	109.5
C10—C12—H12C	109.5	H36B—C36—H36C	109.5
H12A—C12—H12C	109.5	O7—C37—H37A	109.5
H12B—C12—H12C	109.5	O7—C37—H37B	109.5
C7—C13—H13A	109.5	H37A—C37—H37B	109.5
C7—C13—H13B	109.5	O7—C37—H37C	109.5
H13A—C13—H13B	109.5	H37A—C37—H37C	109.5
C7—C13—H13C	109.5	H37B—C37—H37C	109.5
H13A—C13—H13C	109.5	O8—C38—H38A	109.5
H13B—C13—H13C	109.5	O8—C38—H38B	109.5
O4—C14—C15	118.0 (7)	H38A—C38—H38B	109.5
O4—C14—C19	124.3 (6)	O8—C38—H38C	109.5
C15—C14—C19	117.7 (7)	H38A—C38—H38C	109.5
C16—C15—C14	122.4 (8)	H38B—C38—H38C	109.5

Symmetry codes: (i) *x*+1/2, −*y*+1/2, −*z*; (ii) *x*−1/2, −*y*+1/2, −*z*.

### Hydrogen-bond geometry (Å, °)

**Table:**

*D*—H···*A*	*D*—H	H···*A*	*D*···*A*	*D*—H···*A*
N6—H6E···O5^i^	0.86	2.01	2.843 (8)	164
N4—H4E···O2	0.86	2.06	2.873 (8)	157
O7—H7···O4	0.85	2.22	3.066 (14)	179
O8—H8D···O1^iii^	0.82	2.24	3.009 (12)	157

Symmetry codes: (i) *x*+1/2, −*y*+1/2, −*z*; (iii) *x*, *y*+1, *z*.
